# X-ray Absorption (XRA): A New Technique for the Characterization of Granular Activated Carbons

**DOI:** 10.3390/ma14010091

**Published:** 2020-12-28

**Authors:** Jeamichel Puente Torres, Harold Crespo Sariol, Thayset Mariño Peacok, Jan Yperman, Peter Adriaensens, Robert Carleer, Ángel Brito Sauvanell

**Affiliations:** 1 Faculty of Electrical Engineering, Universidad de Oriente, Santiago de Cuba 90600, Cuba; jeamichelp@gmail.com; 2 Provincial Center of Electro-Medicine, Department of Biomedical Metrology, Universidad de Oriente, Santiago de Cuba 90600, Cuba; 3 Applied Acoustic Laboratory, Faculty of Chemical Engineering, Universidad de Oriente, Santiago de Cuba 90600, Cuba; harold@uo.edu.cu (H.C.S.); peacok@uo.edu.cu (T.M.P.); 4 Research Group of Applied and Analytical Chemistry, Faculty of Sciences, Hasselt University, 3590 Diepenbeek, Belgium; peter.adriaensens@uhasselt.be (P.A.); robert.carleer@uhasselt.be (R.C.); 5 Energetic Efficiency Center, Faculty of Chemical Engineering, Universidad de Oriente, Santiago de Cuba 90600, Cuba; albrito@uo.edu.cu

**Keywords:** activated carbon, X-ray absorption, digital image processing, adsorption

## Abstract

The X-ray absorption (XRA) method using digital image processing techniques is a reliable technique to determine the exhaustion degree of granular activated carbons (GACs). Using an innovative digital image processing technique, the identification of individual adsorbed molecules or ions in a GAC was possible. Adsorption isotherm models (Langmuir and Freundlich) were used to simulate the adsorption equilibrium data of Methylene Blue (MB), nickel, cobalt and iodine. Freundlich equation was found to have the highest value of *R*^2^ compared with Langmuir. The identification of distinctive patterns applying XRA for different adsorbed ions and molecules onto GAC was explored. It is demonstrated that unique XRA configurations for each adsorbed ion or molecule are found, as well as a proportional relationship between its incident energy (needed to achieve maximum photon attenuation) and the (effective) atomic number, the adsorbate mass and the molar or atomic mass of adsorbed molecule or ion. XRA method in combination with image histogram modifications was used to obtain a digital signature of adsorbed ions/molecules, giving distinct *GSI* values for each one in the used energy range. Probabilistic models prove that XRA results are within relationships between effective atomic number and photonic interaction probability, reinforcing the potentialities of XRA for monitoring (multi-)ion and/or molecule combinations on GAC using advanced digital image processing techniques. It was proved that the proposed approach could assess different adsorbed ions/molecules onto GACs in water purification systems.

## 1. Introduction

X-ray were discovered by the German physicist Wilhelm K. Röntgen (1845–1923) for which he won the Nobel Prize in 1901. While X-ray have been used for commercial elemental analysis since the 1950s, X-ray spectroscopy is much older than that, dating back to 1909 when Charles G. Barkla found a relationship between X-ray radiating from an element and its atomic weight. In 1913, Henry G.J. Moseley (1887–1915) found a linear relation between the square root of the frequency of X-rays corresponding with the K line transitions in an X-ray spectrum (K-alpha lines) and the atomic number (Z) of 40 elements. He was credited with the revision of the periodic table based on Z rather than the atomic mass (A). He later laid the foundation for identifying elements in X-ray spectroscopy by establishing a relationship between frequency (energy) and the atomic number, a basis of X-ray spectrometry [1]. According to the Rutherford–Bohr model of the atom, electrons orbit around a positive nucleus. Only certain orbital states with specific energies exist, and these are defined by quantum numbers. When increasing Z, orbits are occupied on the basis of minimum energy, those nearest the nucleus, and therefore the most tightly bound, being filled first. Orbital energy is determined mainly by the principal quantum number [2].

The populations of the inner shells are governed by the Pauli exclusion principle, which states that only one electron may possess a unique set of quantum numbers [3]. Valence electrons occupying outer orbits are usually not directly involved in the production of X-ray spectra, which are therefore largely unaffected by chemical bonding. The most intense *K* line is *Kα_1_* (the less intense *Kα_2_* line is usually not resolved, and the combined line is designated *Kα*). The most intense *L* line is *Lα_1_*. Because of the splitting of the *L* shell into three subshells, the *L* spectrum is more complex than the *K* spectrum and contains at least 12 lines, although many of these are weak [1,2,3]. Energies are measured in electron volt (eV), 1 eV being the energy corresponding to a change of 1 V in the potential of an electron (=1.602 × 10^−19^ J). This unit is applicable to both X-ray and electrons. The critical excitation energy (*E_C_*) is the minimum energy needed for bombarding electrons (or other high-energy particles) in order to create an initial vacancy. In electron probe analysis, the incident electron energy (*E_0_*) must exceed E_C_ and should preferably be at least twice *E_C_* to give reasonably high excitation efficiency [1,2]. For atomic numbers above about 35, it is common to change from *K* to *L* lines to avoid the need for an excessively high electron beam energy (which has undesirable implications with respect to the penetration of the electrons in the sample (penetration depth), and in any case may exceed the maximum available accelerating voltage).

However, for the application in this work, penetration power results in a useful tool to acquire the needed information, considering that all previous considerations are done using the photons that pass through the sample. The objective of qualitative analysis is to find which elements are present in an unknown specimen by identifying the lines in the X-ray spectrum using tables of energies or wavelengths. Ambiguities are rare and can invariably be resolved by considering additional lines as well as the main one.

On the other hand, new techniques based on X-ray absorption technology using medical images are getting visibility for the characterization of porous materials. Medical images utilize several different physical principles or image modalities for its formation. An X-ray image obtained from the exposition of matter to X-ray provides a contrast of intervening structures [4] as a direct function of its density (*ρ*) and *Z* (atomic number). The digitalized X-ray image is a grey-scale matrix that can be in different formats depending on the nature of the image. For binary or intensity images, image histogram constitutes a useful tool for analyzing the characteristics of the resultant image, providing important information about the intensity levels and the total pixels in the image [4].

Since X-ray methods have been successfully applied to determine the texture and elemental composition of different materials, the use of advanced digital images processing on digital X-ray radiography (XRA) is an interesting and suitable method to explore for detecting different adsorbed ions and/or molecules and distribution in granular activated carbons (GACs). In this study, two models (Langmuir and Freundlich) were used to describe the sorption process of nickel, cobalt, MB and iodine onto GAC. Furthermore, XRA method was used as an indirect method for determining distinctive patterns for each adsorbed molecule or ion. Interesting relationships between incident energy to achieve maximum photon attenuation, adsorbate mass, (effective) atomic number and atomic mass or molar mass of adsorbed ions or molecules were found, allowing to detect typical grey scale intensity (*GSI*) values for each adsorbed ion or molecule in the analyzed energy range as well as to formulate conclusions about its reliability using probabilistic models. The main purpose of this paper is to prove that the XRA technique can identify (qualitative analysis) and quantify (quantitative analysis) adsorbed ions and molecules on GAC.

## 2. Materials and Methods

### 2.1. Chemicals

**Figure 1:**
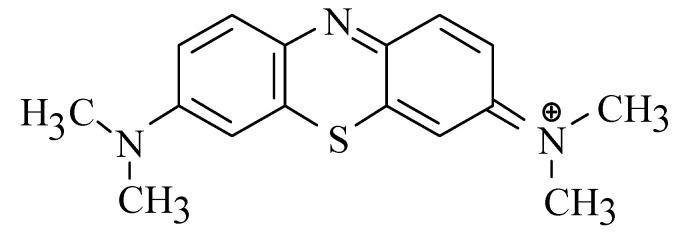
Chemical structure of Methylene Blue ion.

Nickel (Ni) is a transition metal, with atomic number 28 and main oxidation state +II situated in Group 10 of the Periodic Table (at. wt. 58.71). Cobalt (Co) is a ferromagnetic metal, with atomic number 27 and main oxidation states of +II and +III (at. wt. 58.93). Molecular iodine (I_2_) (mol. wt. 253.8) (analytical grade, Merck) was dissolved in water using Kl (analytical grade, Merck KGaA, Darmstadt, Germany) with in situ formation of the triiodide anion (I_3_^−^) (mol. wt. 380.7). Methylene Blue (MB) (analytical grade, Merck KGaA, Darmstadt, Germany) in aqueous solution is present as a positive charged organic ion with molecular formula C_16_H_18_N_3_ClS (mol. wt. 319.85). The chemical structure of MB^+^ ion is shown in Figure 1.

### 2.2. Adsorbent

A commercial granular activated carbon, from Norit (Cabot Norit GmbH, Rheinfelden, Germany), was used as adsorbent. This GAC was studied in the past and its specifications can be found in [5,6]. GAC samples for adsorption were dried at 200 °C during 1 h in a Boxun oven (BGZ Series) applying the ASTM Standard Test Methods D2267-04 for moisture determination. Samples were held overnight in a silica-gel desiccator until being measured.

### 2.3. Preparation of Metal and Dye Solutions

A stock solution of 5 g/L of nickel and cobalt was prepared by dissolving NiSO_4_·6H_2_O (analytical grade, Merck KGaA (Darmstadt, Germany)) and CoSO_4_·7H_2_O (analytical grade, Merck) in Milli-Q water. Different concentrations were obtained by diluting the stock solution (25, 50, 125, 200, 450 and 700 mg/L). Solution pH (pH = 5) was fixed using H_2_SO_4_ (0.5%) (analytical grade, Merck KGaA (Darmstadt, Germany)); 0.3 g (for nickel isotherm) and 0.15 g (for cobalt isotherm) of GAC was added to each flask with 30 mL of solution and shaken for 24 h at room temperature. The concentration of metal ions (Ni and Co) was determined using an inductively coupled plasma spectrophotometer (PerkinElmer Optima 3000 DV ICP-AES (PerkinElmer Inc., Shelton, CT, USA)) with an axial plasma configuration.

A stock solution of 1N iodine was prepared according to the Standard Test Method for determination of iodine number of GAC. All other solutions were prepared by diluting this stock solution (0.05, 0.1, 0.2, 0.4, 0.6 and 0.8 N); 0.15 g of GAC was added to each flask of 30 mL solution and shaken for 24 h at 25 °C. Concentrations were measured by titration using a standardized solution of Na_2_S_2_O_3_ (0.1 N) (analytical grade, Merck).

A stock solution of Methylene Blue (analytical grade, Merck) at a concentration of 5 g/L was prepared; 30 mL of the diluted stock solution (250, 500, 750, 1000, 1500 and 2000 mg/L) was added to different flasks with 0.15 g of GAC and shaken for 24 h at 25 °C. Solution pH (pH = 10) was fixed using 0.1 mol/L of NaOH (analytical grade, Merck). Solution concentrations were determined using a spectrophotometer (Ultrospec, Biochrom Fisher Scientific UK Ltd., Loughborough, UK), at *λ* = 664 nm and a calibration range of 0.5–5 µg/L.

### 2.4. Langmuir Isotherm

Langmuir equation [7] was developed considering the following assumptions: (1) a fixed number of accessible sites is available on the adsorbent surface and all active sites have the same energy; (2) adsorption is reversible; (3) once an adsorbate occupies a site, no further adsorption can occur on that site; and (4) there is no interaction between adsorbate species. Langmuir isotherm model is used to predict the sorption of dissolved molecules/elements onto a solid phase [7,8]. Langmuir model is described in Equation (1) [9]:

(1) $$q_{e}=\frac{q_{m}\cdot K_{L}\cdot C_{e}}{1+K_{L}\cdot C_{e}}$$

where *q_e_* is the amount of adsorbate uptake at equilibrium (in mg/g), *q_m_* is the maximum capacity of adsorption of an adsorbate (in mg/g), *C_e_* is the adsorbate concentration at equilibrium (in mg/L) and *K_L_* is the Langmuir constant related to the affinity between adsorbent and adsorbate (in L/mg).

Limitations in using the Langmuir model by its linear equation have been highlighted recently [9]. The transformation of data for linearization can result in modifications of error structure, introducing an error into the independent variable and altering the weight of each data point, which often leads to differences in the fitted parameter values of the Langmuir model between its linear and nonlinear versions.

Separation factor or equilibrium parameter (*R_L_*) represents an essential tool in order to describe the essential characteristics of the Langmuir isotherm, if Langmuir model adequately describes the experimental data. This parameter is a constant separation factor (dimensionless) of the solid–liquid adsorption system, which is defined as follows [9]:

(2) $$R_{L}=\frac{1}{1+K_{L}\cdot C_{0}}$$

where *R_L_* is Langmuir equilibrium parameter (dimensionless) and *C*_0_ is the initial adsorbate concentration (mg/L).

### 2.5. Freundlich Isotherm

Freundlich isotherm model assumes that the adsorption process takes place on a heterogeneous surface [10]. Freundlich model is one of the earliest empirical equations used to describe equilibrium data. Freundlich nonlinear equation is described in Equation (3) [10].

(3) $$q_{e}=K_{F}\cdot C_{e}{}^{n}$$

where *K_F_* is the Freundlich constant (in mgg(mgL)n) and *n* is the Freundlich intensity parameter (dimensionless).

The parameter n in Freundlich model indicates the magnitude of the adsorption driving force or the surface heterogeneity [9,10].

### 2.6. Effective Atomic Number

When dealing with a molecule or a mixture of molecules, it is convenient to describe this system by an effective atomic number (*Z_eff_*). In the energy range where Compton absorption is important, the absorption is independent of *Z* and calculation of *Z_eff_* is unnecessary. The energy region of interest when discussing *Z_eff_* is then from 20 to 100 keV [11], where the photoelectric process is dominant over the Compton process [11,12] and radiologic machines are equipped to work. Calculation of *Z_eff_* of the MB ion for total photon interaction was carried out using Equation (4) [11].

(4) $$Z_{eff}=\sqrt[{m}]{a_{1}Z_{1}{}^{m}+a_{2}Z_{2}{}^{m}+\ldots +a_{z}Z_{z}{}^{m}}$$

Coefficients *a_i_* can be defined using the following equation:

(5) $$a_{i}=\frac{\frac{N_{A\;}\cdot \;Z_{i}\;\cdot \;w_{i}}{A_{i}}}{\sum_{i}\frac{N_{A\;}\cdot \;Z_{i\;}\cdot \;w_{i}}{A_{i}}}\;\mathrm{for}\;i\;=\;1,\;z$$

where *N_A_* is the Avogadro number, *Z_i_* is the atomic number of element *i*, *A_i_* is the atomic weight of element *i*, *w_i_* is the weight proportion of element *i* (dimensionless) and *m* is the effective dependency of photoelectric effect in the energy range used (20–100 keV).

Mayneord [9] first introduced *Z_eff_* and recommended an exponent *m* = 2.94. The determination of *Z_eff_* involves considerable effort; theoretical calculations should be verified considering experimental data. Weight proportions for each constituent element of MB ion (see Section 2.1) were calculated online using XCOM software version 1.0 (NIST Technologies, Gaithersburg, MD, USA) [13,14]. When an MB molecule is dissolved in aqueous solution, a complete dissociation takes place into MB^+^ and/or MB^2+^ depending on the pH. At pH = 10, MB ion will be present only as MB^+^. Table 1 depicts weight proportions values, atomic number and atomic weight for each element of the MB ion for calculating its *Z_eff_*.

*Z_eff_* of the MB ion in the energy range used (20–100 keV) was calculated using the values showed in Table 1 and Equation (5) being approximately 9. For the iodine molecule, *Z_eff_ = Z*.

### 2.7. X-ray Digital Radiography Experiments (XRA)

X-ray radiography experiments were performed at seven different energies for each analyzed ion and molecule. Six *q_e_* values (see Table 2) were analyzed using X-ray radiography. *GAC* charged with Ni, Co and MB were analyzed using 22, 27, 33, 40, 50, 60 and 70 keV, respectively. Energies above 50 keV caused an increase in the penetrating power of the incident photon beam; as more photons pass through the material, darker images are obtained with fewer differences in *GSI* values, making the experiment useless for Ni, Co and MB. *GAC* charged with iodine were analyzed using 40, 50, 60, 70, 80, 90 and 100 keV, respectively. Whether images were obtained for *GAC* charged with iodine using energy values below 40 keV, as only a minor amount of photons passes through the material, no significant differences in *GSI* values were detected for the analyzed samples. When energies above 150 keV were used to analyze *GAC* iodine samples, darker images were obtained and it was not possible to detect significant differences in *GSI* values between samples. Table 2 depicts *q_e_* and *C_e_* values for *GAC* charged with, respectively, Ni, Co, MB and *I*_2_ to develop X-ray experiment.

Experiments applying energies between 22 and 33 keV (related to penetration depth) were conducted at 125 mAs (related to the amount of X-ray reaching the sample) [15] using a TOSHIBA Mamorez-mgu 100d, TOSHIBA, Tokyo, Japan. X-ray equipment used for mammography studies in manual mode (the energy of incident X-ray photons needed to be adjusted manually). X-ray experiments applying energies between 40 and 100 keV were conducted using 40 mAs [15] in a TOSHIBA KXO-36 s (used for general clinical radiography). X-ray radiography experiments were performed according to the methodology described in [15]. XRA experiments and data processing of the X-ray digital images were performed following the images processing algorithm applied in [15,16]. The spatial concentration (*S_C_*) (in pixel), for a specific grey-scale intensity level (*GSI*) was related with the normalized grey scale value when the total number of pixels maximizes, i.e., *T_P_*(*GSI*) [15,16]. The mathematical analysis of the digital X-ray radiographic images was performed using dedicated software developed in MATLAB^®^ 2015 specifically for this application. A focal distance of 60 cm and a sample thickness of 1.2 cm were used for all the experiments.

### 2.8. Relationship between Grey-Scale Intensity (GSI) and Mass Attenuation Coefficient Using XRA. Spatial Concentration (S_C_)

Since photoelectric effect predominates for the voltage range used for clinical X-ray radiography (20–120 keV), photon attenuation can be expressed using the formula presented in Equation (6) [11].

(6) $$N=\;N_{0}\cdot e^{-\mu .x}$$

where *N* is the intensity or number of attenuated photons (dimensionless), *N*_0_ is the initial Intensity or number of incident photons (dimensionless), *µ* is the linear absorption coefficient (in cm^−1^) and *χ* is the sample thickness (in cm).

According to recent publications [15,16], it is possible to establish a mathematical relationship between attenuated photons (*N*) and *GSI* using the Equation (7) [15].

(7) GSI=f(N)

According to Equation (8), it is possible to express *GSI* as an equivalent exponential function of the initial grey-scale intensity (*GSI*_0_) using Equation (8) [15].

(8) $$GSI=GSI_{0}\cdot e^{\mu .x}$$

The mass attenuation coefficient (ƫ) is obtained from the linear absorption coefficient (*µ*) divided by the density of the material (*ρ*). This coefficient is independent of the material density [9]. The grey-scale intensity of incident photons (*GSI*_0_) was calculated according to the method in [15]. The density of the virgin adsorbent used (*GAC*) is approximately 0.38 g/cm^3^. Relative density values of each ion or molecule (Ni, Co, MB and *I*_2_) (*ρ_i_*) were calculated using *q_i_* and the density of the virgin adsorbent GAC as shown in Equation (9).

(9) $$\rho_{i}=\;0.38\cdot q_{i}$$

where *ρ_i_* is the relative density of adsorbed ion or molecule *i* (in g/cm^3^) and *q_i_* is the ratio between amount of adsorbate uptake at equilibrium and maximum capacity of adsorption of an adsorbent (dimensionless).

Equation (8) can be reorganized as:

(10) $$\mu =\frac{ln\left(\frac{GSI}{GSI_{0}}\right)}{x}$$

Using Equation (10), it is possible to express the mass attenuation coefficient as a function of the grey-scale intensity (Equation (11)).

(11) $${ƫ}_{i}=\frac{\mu }{\rho_{i}}=\frac{ln\left(\frac{GSI}{GSI_{0}}\right)}{\rho_{i}\cdot x}$$

where ƫ*_i_* is the mass attenuation coefficient of ion or molecule *i* (in cm^2^/g).

An increase in the X-ray energy causes a decreasing trend in *GSI* values [15,16]; at the same time, considering Equation (10), when *GSI* values tend to decrease, the mass attenuation coefficient should decrease in a proportional way.

XRA method uses digital image processing on digital X-ray radiography. An X-ray beam of variable energy between 20 and 120 keV, a sample thickness of 12 mm and a focal distance of 60 cm were used for all the experiments. The XRA experiments and data processing of the X-ray digital images were performed following the image processing algorithm applied in [15]. The spatial concentration *S_C_* (in pixel) for a specific *GSI* level was related with the normalized grey scale value when the total number of pixels is maximized, i.e., *T_P_(GSI)*, and can be expressed by Equation (12) [15,16].

(12) $$S_{C}\left(GSI\right)=T_{P}\left(GSI\right)$$

where *S_C_(GSI)* is the spatial concentration of the intensity level *GSI* (in pixel).

The mathematical analysis of the digital X-ray radiographic images was performed using dedicated software developed in MATLAB^®^2015 (MathWorks, Natick, MA, USA) specifically for this application.

### 2.9. Relationship between Energy (to Achieve Maximum Photon Attenuation), Adsorbate Mass, Molar Mass and Atomic Number

The relative concentration of an ion or molecule *i* adsorbed by GAC can be mathematically expressed using Equation (13).

(13) $$C_{i}=\frac{m_{i}}{m_{T}}$$

where *C_i_* is the relative concentration of ion or molecule *i* (mg/g), *m_i_* is the mass of ion or molecule *i* adsorbed (in mg) and *m_T_* is the total mass of sample studied (mass of ion or molecule adsorbed and mass of adsorbent) (g).

Global density *ρ_T_* can be calculated using Equation (14):

(14) $$\rho_{T}=\frac{m_{T}}{V_{T}}$$

where *V_T_* is the total solid sample volume in the cuvette (in cm^3^).

According to Equations (13) and (14), it is possible to express the relative concentration of ion or molecule *i* using Equation (15).

(15) $$C_{i}=\frac{m_{i}}{\rho_{T}\cdot V_{T}}$$

Considering the shape of the X-ray cuvette, the total volume *V_T_* can be mathematically expressed using Equation (16).

(16) $$V_{T}=a\cdot b\cdot x$$

where *a* is X-ray cuvette depth (in cm), *b* is X-ray cuvette width (in cm) and *x* is X-ray cuvette height or sample thickness (in cm).

Substituting Equation (16) into Equation (15) and assuming *a* = *b* = 1, considering that changes in *a* and *b* cause proportional changes in *m_i_* and *C_i_*, it is possible to obtain Equation (17):

(17) $$C_{i}=\frac{m_{i}}{\rho_{T}\cdot x}$$

Molar mass or atomic mass (*M_i_*) can be used as a conversion factor between mass *m_i_* (in mg) and amount of substance *o_i_* (in mmol) of ion or molecule *i*:

(18) $$m_{i}=o_{i}\cdot M_{i}$$

Substitution of Equation (18) into Equation (17) gives:

(19) $$C_{i}=\frac{o_{i}\cdot M_{i}}{\rho_{T}\cdot x}$$

The behavior of the photoelectric effective section with the energy changes for energies below or above 0.5 MeV. For energies above 0.5 MeV, electron behavior is relativistic and photoelectric effective section decreases inversely proportional to the energy (*hv*) [10]. A global description of the photoelectric effective section or interaction probability for a specific ion or molecule for energies below 0.1 MeV (between 20 and 100 keV) is given by Equation (20) [11,12,13,14].

(20) $$P_{i}\sim \frac{Z_{i}{}^{k}}{E^{l}}$$

for 4 < *k* < 5 and 1 < *l* < 3.5 [11], where *P_i_* is the photoelectric interaction probability of element *i* (dimensionless), *Z_i_* is the (effective) atomic number of ion or molecule *i* (dimensionless), *E* is the energy of the incident photon beam (in keV), *k* is the effective dependency of photoelectric effective section with (effective) atomic number *Z* (dimensionless) and *I* is the effective dependency of photoelectric effective section with energy *E* (dimensionless).

For low (effective) atomic number elements/molecules, it is recommended to use *k* = 4 and *l* = 3 [12,13,14]. Cunningham [11] suggested using *k* = 4.8 and *l* = 3.5 for elements/molecules with (effective) atomic number equal to or below 50. Considering that an increase in the relative concentration of the analyzed ion/molecule (*i*) causes an increase for atoms/molecules with atomic or effective atomic number *Z_i_*, Equation (20) can be rewritten as:

(21) $$P_{i}\sim o_{i}\frac{Z_{i}{}^{k}}{E^{l}}$$

Using Equation (19) in Equation (21), one obtains:

(22) $$P_{i}\sim \frac{C_{i}\cdot x\cdot \;\rho_{T}}{M_{i}}\cdot \frac{Z_{i}{}^{k}}{E^{l}}$$

Assuming the maximum interaction probability (*P* = 1) for a specific ion or molecule *i*, we obtain:

(23) $$1\sim \frac{C_{i}\cdot x\cdot \rho_{T}}{M_{i}}\cdot \frac{Z_{i}{}^{k}}{E^{l}}$$

Which allows defining *E* as:

(24) $$E\sim \sqrt[{l}]{C_{i}\cdot x\cdot \frac{Z_{i}{}^{k}\cdot \rho_{T}}{M_{i}}}$$

From substitution of Equation (17) into Equation (24), we obtain:

(25) $$E\sim \sqrt[{l}]{m_{i}\cdot \frac{Z_{i}{}^{k}}{M_{i}}}$$

Adsorption amount of an ion or molecule *i* (*m_i_*) adsorbed per gram of adsorbent (*m_adsb_*) as defined by Langmuir or Freundlich isotherm can be described using Equation (26) [9,10].

(26) $$q_{e}=\;\frac{m_{i}}{m_{adsb}}$$

Multiplying *q_e_* parameter by the density of the used adsorbent, it is possible to obtain the adsorbate concentration (*C_i_*) expressed as mass–volume relation in the solid phase using Equation (27):

(27) $$q_{e}.\;\rho_{adsb}=\frac{m_{i}}{m_{adsb}}\cdot \rho_{adsb}=\frac{m_{i}}{V_{T}}$$

Rearranging Equation (27) allows the calculation of the adsorbate mass using Equation (28).

(28) $$m_{i}=\;q_{e}\cdot \rho_{adsb}\cdot V_{T}$$

The adsorbent density value (*ρ_asd_*) is constant (0.38 g/cm^3^, Section 2.9); the volume of analyzed samples is also constant, and it has an approximate value of 1.2 cm^3^ [15]. Equation (25) describes a proportional relationship between energy (keV) and atomic or effective atomic number, molar mass and the mass of ion or molecule *i*. This means that the energy value needed to achieve maximum photon attenuation (pixels in “1” in XRA images) is proportional with the amount of adsorbate that can be adsorbed by a determinate mass of GAC or other absorbent material in a fixed volume. Taking into account the above and using the experimental data, it could be possible to calculate a physical constant able to directly describe the relation between energy, atomic or effective atomic number, molar mass and adsorbate mass.

### 2.10. Probabilistic Approach for Comparison between Elements/Molecules

Considering that Equation (25) describes a proportional relationship and not an equality, it was necessary to verify the reliability of this mathematical expression. Values of exponent *k* and *l* can change in function of the energy range used; however, those values should present limited variations between 20 and 120 keV (energy range used for the experiment) with a small error range compared to the values reported in the literature [11,12,13,14]. Using Equation (25), it is possible to develop a probabilistic comparison between optimal energy values (*E*_1_ and *E*_2_) found for analyzed ions or molecules using Equation (29).

(29) $$\frac{E_{1}}{E_{2}}\approx \frac{\sqrt[{l}]{m_{1}\cdot \frac{Z_{1}{}^{k_{1}}}{M_{1}}}}{\sqrt[{l}]{m_{2}\cdot \frac{Z_{2}{}^{k_{2}}}{M_{2}}}}$$

where *E*_1_ and *E*_2_ are energy values found for the best correlation fit between *S_C_* and *q_e_* for ions or molecule 1 and 2 (in keV).

Using calculated values of adsorbate mass as well as energy values found for the best correlation fit between *S_C_* and *q_e_* for ions or molecule 1 and 2, atomic or effective atomic number, molar mass or atomic mass of analyzed molecule or ion, it is possible to obtain a 3D representation of the relation between ions or molecules, keeping exponent *l* constant and analyzing the behavior of the exponent *k* within the range proposed in the literature (in our case, between 4 and 5). If found energy values are correct, the exponent parameter *k* should have an approximate numerical value for the values proposed in the literature [11] for each analyzed ion or molecule and in accordance with the atomic or effective atomic number. The relation between the ratio of energy values (*E*_1_*/E*_2_) found for each ion or molecule 1 and 2 should be the same within a small error range.

## 3. Results and Discussion

### 3.1. Isotherms for the Sorption Process

Relationship between *C_e_* and *q_e_* can be used to evaluate the effectiveness of different models to describe experimental data [9].

#### 3.1.1. Langmuir Isotherms

The nonlinear fitting of the experimental data by the Langmuir isotherm gives not that convincing results in view of their *R*^2^ values, except for Ni. The values of *K_L_*, *q_m_* and *R*^2^ from the correlation between *q_e_* and *C_e_* are presented in Table 3.

Table 4 depicts the linear fitting results for the Langmuir relationship *C_e_*/*q_e_* vs. *C_e_* for each adsorbed ion or molecule.

**Figure 2:**
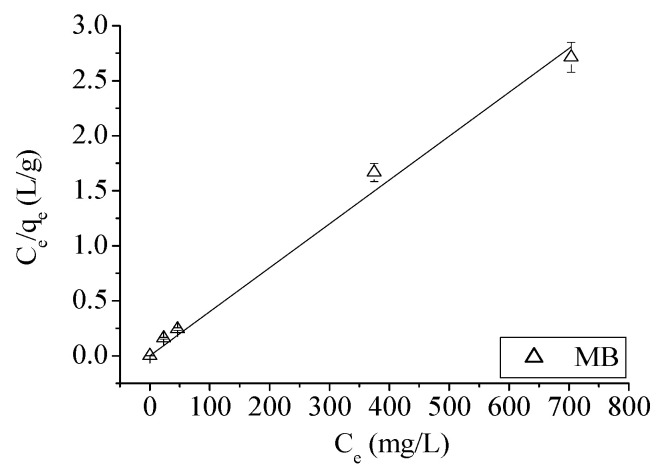
*C_e_*/*q_e_* vs. *C_e_* relation for MB.

Now, only for MB and not for Ni, a good fitting result is found between *C_e_*/*q_e_* and *C_e_*, as shown in Figure 2.

For the other ions and molecule, as demonstrated by Table 4, no good Langmuir linear fitting value for *R*^2^ is obtained. Additionally, *K_L_* values are totally different from each other using both approaches (Table 3 and Table 4). On the contrary, *q_m_* values are comparable but *R*^2^ values are totally different. Error propagation on both parameters *K_L_* and *q_m_* behave complete differently according to which fitting approach is applied. This indicates that the obtained linear and nonlinear Langmuir fittings are not acceptable and are not suitable to describe the adsorption for these ions and molecule, certainly also in view of the very good Freundlich fitting results obtained. Nevertheless, Table 5 depicts the separation factor calculated for each adsorbed ion or molecule [9].

A separation factor between 0 and 1 indicates favorable adsorption [9]. According to Table 5, separation factors between 0 and 1 are found for nickel, cobalt, MB and iodine, indicating a favorable adsorption of all those ions or molecule onto GAC.

#### 3.1.2. Freundlich Isotherms

**Figure 3:**
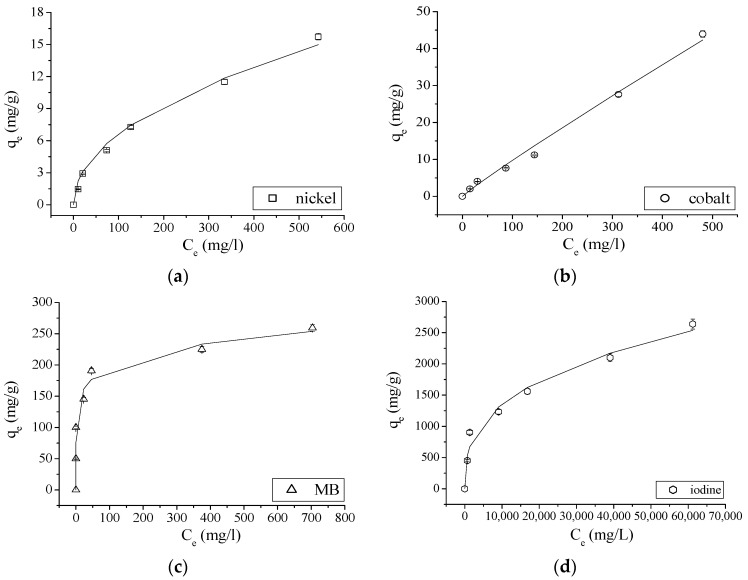
Adsorption isotherms for Freundlich model of: nickel (**a**); cobalt (**b**); MB (**c**); and iodine (**d**) onto GAC at 25 °C.

Equilibrium adsorption data of nickel, cobalt, MB and iodine onto GAC was tested with Freundlich isotherm model. Nonlinear fitting graphs for Freundlich isotherm model at room temperature (25 °C) are shown in Figure 3.

The values of *n* and *R*^2^ for the nonlinear fitting between *q_e_* and *C_e_* for Freundlich adsorption isotherms are presented in Table 6a.

When applying linear fitting of the Freundlich model on obtained experimental data, the results presented in Table 6b are obtained.

As could be expected (different propagation behavior of experimental errors using experimental data), slightly different values for the two parameters are found. According to the *R*^2^ values shown in Table 6a,b, the Freundlich adsorption model describes the adsorption of analyzed ions and molecule onto GAC perfectly. The value of the parameter *n* = 1 for cobalt, in the case of a nonlinear fitting, indicates that the adsorption process is linear [9,10], as demonstrated in Figure 3b. On the other hand, linear fitting presents a value of *n* < 1 for all ions and molecule, indicating that the adsorption process is favorable. *K_F_* and *n* values are now comparable in value when using both fitting approaches. However, nonlinear fitting results have to be preferred as experimental error structure is differently modified in both approaches making linear fitting results questionable [9]. Based on *K_F_* values, the adsorption of MB is much faster than the one for *I*_2_ (a factor two faster). Compared to the others, adsorptions for Ni and Co ions are very slow. This agrees with the steep exponential shape of MB and *I*_2_ isotherms compared to the one of Ni and certainly with the linear shape of Co isotherm.

### 3.2. X-ray Absorption (XRA)

**Figure 4:**
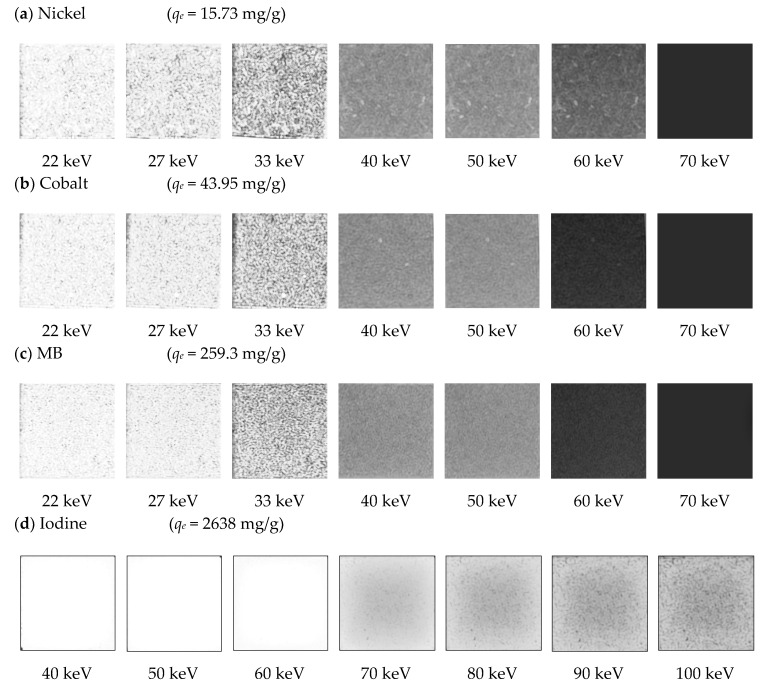
Digital radiographies taken at different energies for each adsorbed molecule/element onto GAC using different *q_e_* values: (**a**) for nickel; (**b**) for cobalt; (**c**) for MB and (**d**) for iodine.

X-ray digital radiographic data at each analyzed energy of incident photon beam were stored and independently examined to acquire the needed information to determine if significant differences can be found between different GAC samples loaded with different adsorbed ion/molecules. Figure 4 depicts digital radiographies (using XRA) of GAC loaded with the highest adsorbate mass of nickel (*q_e_* = 15.73 mg/g), cobalt (*q_e_* = 49.95 mg/g), MB (*q_e_* = 259.3 mg/g) and iodine (*q_e_* = 2638 mg/g) (see Section 2.3) using 22, 27, 33, 40, 50, 60 and 70 keV for nickel, cobalt and MB and 40, 50, 60, 70, 80, 90 and 100 keV for iodine.

An increase in energy causes a decrease in the grey-scale level obtained, due to a proportional increase of the penetration power of the incident photon beam. The obtained grey-scale level is proportional to the nature of the adsorbed molecule or ion and the number of pixels in that particular grey-level (spatial concentration, *S_C_*) and is also related with its adsorbed amount [15,16], keeping the sample thickness constant between experiments [15]. Higher *q_e_* values cause a higher amount of wither pixels (pixels at “1”) at the same irradiation energy, however a decreasing trend in the grey-scale values is observed with increasing energy of the incident beam. According to Figure 4, it is not possible to observe changes in the grey-scale radiographic images for nickel using energies values above 60 keV (see *S_C_* values in Table 7) for *q_e_* = 15.73 mg/g. Cobalt shows a similar behavior, with no variations in the normalized grey-scale above 60 keV, with *q_e_* = 43.95 mg/g. Nickel and cobalt present almost equal atomic numbers (*Z* = 28 and *Z* = 27, respectively); thus, the grey-scale changes seen in radiographic images are mainly caused by adsorbed mass differences between the ions. MB also depicts a similar pattern (but not equal) with respect to nickel and cobalt when radiographic images are compared; the calculated *Z_eff_* for MB is the lowest of all analyzed ion/molecules (*Z_eff(MB)_* = 9). However, its *q_e_* value is 16 times higher in comparison with nickel and six times higher when compared with cobalt. There are no detectable changes in the grey-scale (*q_e_* = 259.3 mg/g) when energies above 60 keV are used. Its effective atomic number proves that *Z* or *Z_eff_* cannot be used as a single fundamental parameter to describe differences. However, the adsorbed mass used for this ion is significantly higher than the adsorbate mass of nickel and cobalt onto GAC. No significant differences between iodine samples are found using energies values under 40 keV. At the same time, it is not possible to obtain a completely darker image for analyzed iodine samples. Considering the high atomic number and very high adsorbed mass values used for the adsorption study of iodine, it will take energy values above 150 keV to achieve zero attenuation and obtain a completely dark image. X-ray equipment for medical diagnosis actually installed in hospitals cannot be used, since the maximum energy is usually 120 keV. In Figure 4, high grey-scale values can be observed for iodine using high-energy values up to 100 keV. Spatial concentration (*S_C_*) [15] of pixels at “1” in the normalized grey-scale was correlated with *q_e_* for each molecule or ion, at each used energy. Table 7 depicts *q_e_* and *S_C_* values for each used energy.

**Figure 5:**
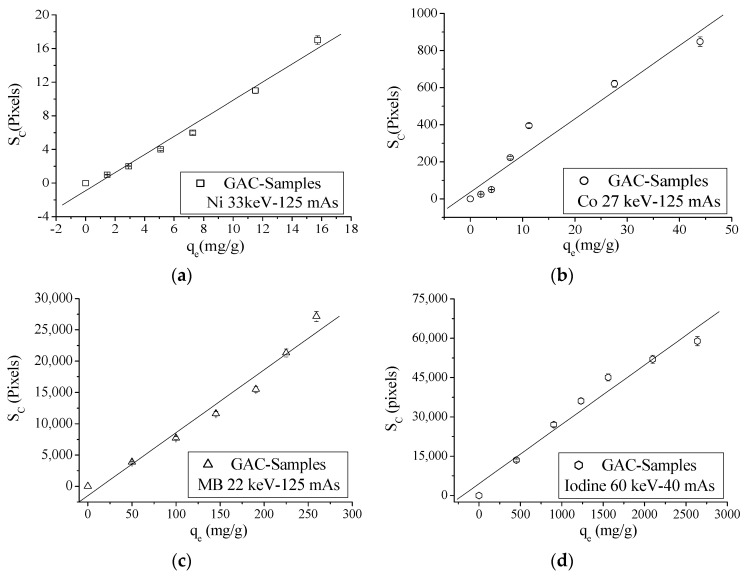
Correlation graphs between *S_C_* and *q_e_* values: (**a**) nickel; (**b**) cobalt; (**c**) MB; and (**d**) Iodine for virgin GAC and ion or molecule loaded GAC samples.

For energy values between 22 and 33 keV, an initial intensity of 125 mAs was used. For energies above 40 keV an intensity of 40 mAs was used for all experiments [15]. In Table 7, for energy values above 33 keV, no pixels in “1” can be noticed for nickel and cobalt. For MB, a similar trend for energies above 27 keV can be observed. The same phenomena can be seen for iodine for energy values above 60 keV. The spatial concentration (*S_C_*) is correlated with *q_e_* values in order to find the energy value where the best correlation between *S_C_* and *q_e_* can be found for each adsorbed molecule or ion. Figure 5 depicts the correlation graphs between *S_C_* (XRA) and *q_e_* values of different adsorbed ions/molecule onto GAC at different energies.

Correlation graphs between *S_C_* and *q_e_* values of analyzed ions or molecules evaluating other used energies are shown in Figure S1. Correlation parameters are presented in Table 8.

According to Table 8, good correlation coefficients can be found between *S_C_* and *q_e_* when an optimal energy for incident photons is used. For nickel, a fitting goodness of 0.99 is found at 33 keV and 0.98 at 27 keV, both at 125 mAs. According to Equation (25), the optimal energy for this ion (for *q_e_* variations between 1.46 and 15.73 mg/g) should be between 20 and 40 keV. For *c* = cobalt, the best fitting result of 0.97 using 27 keV is noticed. According to Equation (25) and calculating the estimated adsorbed mass of cobalt from *q_e_*, the optimal energy values to describe the adsorption of cobalt onto GAC should be approximately between 26 and 51 keV (*q_e_* within the range of 2.01–43.95 mg/g). MB shows the best correlation parameters only at 22 keV and 125 mAs, with a 0.98 of fitting goodness. For this ion, optimal energy to describe its adsorption onto GAC should be between 16 and 24 keV for found *q_e_* values (between 49.97 and 259.3 mg/g) using Equation (25). Iodine is the adsorbed molecule with the highest atomic number (*Z* = 53), and it is also the compound with highest values of *q_e_*. For this molecule, the best correlation parameter between *S_C_* and *q_e_* is only found for 60 keV and 40 mAs with a 0.98 fitting goodness. Predicted energy values for *q_e_* values between 451.5 and 2638 mg/g are approximately between 50 and 74 keV using Equation (25).

**Figure 6:**
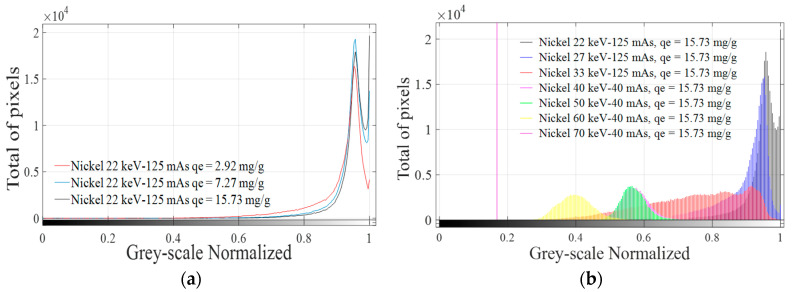
Combination of image histograms of nickel radiographies at: different *q_e_* values and constant energy (22 keV) (**a**); and different energies at the same *q_e_* value (15.73 mg/g) (**b**).

Digital image histograms obtained after the application of XRA technique to each analyzed sample were calculated using MATLAB^®^2015. A combination process of those histograms was applied to obtain an absorption signature for each adsorbed molecule or ion in the studied energy range. A combination of histograms is shown in Figure 6 using nickel as an example at different *q_e_* values keeping the energy constant (Figure 6a) and changing the energy range between 22 and 70 keV while keeping *q_e_* constant (Figure 6b).

**Figure 7:**
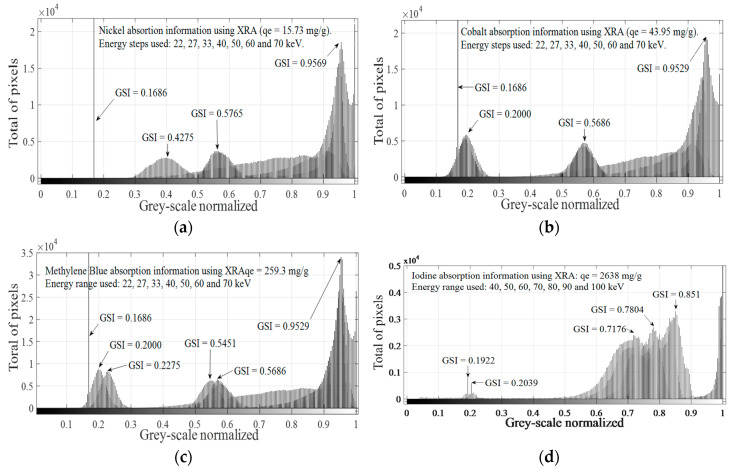
Histogram combined strategy using XRA data for: nickel (**a**); cobalt (**b**); MB (**c**); and Iodine (**d**).

According to Figure 6a, the same pattern is found for different values of adsorbed mass using *q_e_* values, despite significant changes in the spatial concentration of pixels in “1”, as can be seen in Table 7. Significant differences for pixels at “1” are more difficult to detect when *q_e_* increases, making the judgment of resolution difficult. Increasing the penetration power could be a solution for bandwidth problems, which is narrower with increasing *q_e_* values. Doing this and considering that the same pattern is obtained when different adsorbed masses are evaluated, for a fixed selected *q_e_* value (e.g., *q_e_* = 15.73 mg/g), image histograms obtained at each irradiation energy are combined in one plot, as shown in Figure 6b. The method previously explained was applied for all explored ions and molecule identifying characteristics peaks for each adsorbed ion or molecule in the energy range used. Considering that changes in *q_e_* only cause changes in the spatial concentration and not in the grey-scale level, maximum *q_e_* values for each adsorbed molecule or ion were selected and the combination process, as displayed in Figure 6b, was applied for all GAC samples, as shown in Figure 7, at each irradiation energy.

In Figure 7, it is possible to observe different patterns for each analyzed molecule or ion. Nickel presents distinctive peaks at *GSI* = 0.1686, *GSI* = 0.4275, *GSI* = 0.5765 and *GSI* = 0.9569. Cobalt shows typical peaks at *GSI* = 0.1686, *GSI* = 0.2000, *GSI* = 0.5686 and *GSI* = 0.9529. MB shows typical peaks at *GSI* = 0.1686, *GSI* = 0.2000, *GSI* = 0.2275, *GSI* = 0.5451, *GSI* = 0.5686 and *GSI* = 0.9529. For MB, four *GSI* values matched with *GSI* values of cobalt (*GSI* = 0.1686, *GSI* = 0.2000, *GSI* = 0.5686 and *GSI* = 0.9529), but with different total pixel values, and only one matched with nickel (*GSI* = 0.1686). The *GSI* peak at 0.1686 is a very narrow peak with a very high maximum total pixel value of 115,000. Iodine depicts an absorption signature slightly different in comparison with nickel, cobalt and MB. In this case, higher *GSI* values are found for each irradiation energy, which is consistent with iodine high atomic number and larger *q_e_* values found for its adsorption onto GAC1. For this molecule, characteristic peaks are found at *GSI* = 0.1922, *GSI* = 0.2039, *GSI* = 0.7176, *GSI* = 0.7804 and *GSI* = 0.8510. Figure 7 shows different patterns for different adsorbed molecule or ions onto GAC1 by the XRA method. Typical *GSI* values were found for each analyzed ion or molecule in the used energy range, which can be used as a digital signature for each adsorbed molecule or ion using the histograms combination strategy. For instance, if more than four peaks are found in the histogram combination figure of *GSI* for MB and Co, *GSI* peaks at 0.2275 and at 0.5451 can be used to prove the presence of MB in a solution instead of Co. For Ni and *I*_2_, differentiation is easier since the pattern of their combined *GSI* peaks is unique and characteristic.

### 3.3. Probability Approach

**Figure 8:**
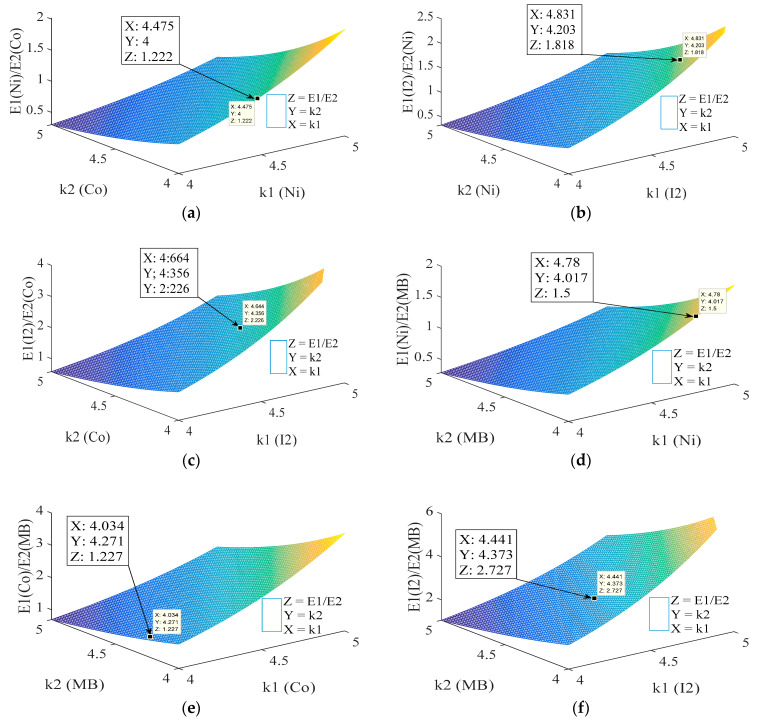
3D plots for comparison: nickel vs. cobalt (**a**); iodine vs. nickel (**b**); iodine vs. cobalt (**c**); nickel vs. MB (**d**); cobalt vs. MB (**e**); and iodine vs. MB (**f**).

To verify the reliability of the application of obtained model (Equation (25)) to describe the photon absorption of adsorbed molecule or ion onto GAC, a probability model (Equation (29)) was developed to verify if experimental values are in concordance with the literature. However, Equation (25) is a proportional equation expressed in terms of keV. Therefore, testing this model and its ability to describe the experimental data obtained are required. To develop this test, a comparison between experimental data and proposed model is done using Equation (29). The ratio of energy values (*E*_1_ and *E*_2_) for a pair of ions or molecules or an ion and molecule obtained experimentally using XRA experiments (left half of Equation (29)) is compared to the ratio of corresponding energy values obtained using Equation (25) (right half of Equation (29)). If the obtained model is accurate to describe obtained data, the error between the ratio of energy values obtained experimentally and by calculation should be small. Equation (29) gives the possibility to evaluate the relationship between the ratio of energy values of two ions or molecules or between an ion and molecule and its effective dependency of photoelectric effective section. This evaluation allows obtaining 3D graphs for studying changes in the effective dependency of photoelectric effective section in order to see if obtained values are within the range reported in the literature [11,12,13,14]. Figure 8 depicts 3D plots of developed model.

Figure 8 shows 3D plots where the relationship between the ratio of obtained energies (energy with best correlation parameters between *S_C_* and *q_e_*) for each adsorbed ion or molecule is shown on the z-axis. The x- and y-axes represent the effective dependency of photoelectric effective section (*k*) with effective atomic or atomic number for each adsorbed molecule or ion. In this probability comparison, the effective dependency of photoelectric effective section with energy (*l*) remains constant (3.5) and *k* is between 4 and 5, except for MB, where *k* = 4 and *l* = 3 (see Equation (29)), mainly caused by the low effective atomic number of MB. According to the literature, ions or molecules with low atomic number (or effective atomic number) should be analyzed using the lowest values of *k* and *l* [11,12]. For a comparison between ions/elements, the ratio of obtained energies where the best correlation is found between *S_C_* and *q_e_* is used. For nickel and cobalt (Figure 8a), a value of approximately 1.222 is found while the experimental result is around 1.222 (= 33 keV/27 keV). Obtained error is approximately 0.0002 (around 0.02%). Values of *k* for both elements are within reported range in the literature being between 4 and 5:4.5 for nickel (*k*_1_) and 4 for cobalt (*k*_2_) approximately. Considering that the differences between nickel and cobalt are mostly caused by differences in adsorbed mass, similar values for the effective dependency of photoelectric effective section are expected because of comparable atomic numbers. In Figure 8b, a comparison between iodine and nickel is presented. According to experimental data, the relationship between the ratio of energies (60 keV/33 keV) is 1.8181. Using the probability approach, a value of 1.818 (*E*_I2_/*E*_Ni_) is obtained, representing an error of 0.01% with respect to experimental results. The effective dependency of photoelectric effective section with atomic number is within a reported range from 4.8 (*k*_Ni_) to 4.2 (*k*_I2_) for iodine and nickel, respectively. The same procedure was used comparing iodine vs. cobalt, nickel vs. MB, cobalt vs. MB and iodine vs. MB. In the first case (Figure 8c), an error of 0.4% with respect to experimental data is found when energies values are compared, with values for *k* within the reported range (approximately 4.6 for *I*_2_ and 4.3 for Co) [11,12,13,14]. When MB and nickel (Figure 8d) are compared, *k* and *l* should be modified for MB, considering its low effective atomic number. After modification, a deviation from experimental values of 0.2% is obtained when obtained energies are compared while *k* values (4.8 for MB and 4.1 for Ni) are within the expected range. The same procedure was applied for the comparison cobalt vs. MB and iodine vs. MB, obtaining errors of 0.2%, respectively, for *k* values around 4 and 4.3 (Co vs. MB) and 4.4 and 4.3 (*I*_2_ vs. MB).

## 4. Conclusions

The adsorption process of nickel, cobalt, Methylene Blue and iodine onto GAC was best described by the Freundlich isotherm.

A proportional dependency was found between incident energy to achieve maximum photon attenuation and adsorbed mass, effective atomic number or atomic number and molar or atomic mass on GAC loaded with nickel, cobalt, MB and iodine.

XRA results were used in combination with image histograms to obtain a digital signature of adsorbed ions or molecules onto GAC. Distinct *GSI* values were found for each adsorbed molecule or ion within the used energy range. It was proved that differentiation can be made between adsorbed ions or molecules using this technique.

Experimental results were compared with probability models, indicating that experimental data obtained are within reported values in the literature [11].

Finally, it was demonstrated that XRA has potential for monitoring (multi-)ion and/or molecule(s) combinations on GACs using advanced digital image processing techniques.

## Figures and Tables

**Table 1:** Weight proportion values, atomic number and atomic weight for each constituent element of MB ion calculated using XCOM software.

Element	*W*	*Z*	*A*
H	0.056722	1	1
C	0.600817	6	12
N	0.131372	7	14
S	0.100251	16	32

**Table 2:** *q_e_* and *C_e_* values for GAC samples charged with Ni, Co, MB and *I*_2_.

Nickel		Cobalt		Methylene Blue		Iodine	
*C_e_*	*q_e_*	*C_e_*	*q_e_*	*C_e_*	*q_e_*	*C_e_*	*q_e_*
0	0	0	0	0	0	0	0
10.4	1.46	14.9	2.01	0.0395	49.97	665	451
20.8	2.92	29.8	4.03	0.079	99.94	1330	903
74.05	5.09	86.85	7.62	23.22	145.31	9077	1232
127.3	7.27	143.9	11.21	46.36	190.68	16,825	1561
335	11.51	312.15	27.58	374.93	224.99	39,036	2099
542.7	15.73	480.4	43.95	703.5	259.3	61,248	2638

$q_{e}=\frac{C_{0}-C_{e}}{m_{adsorbent}}V_{sol}$ (*V_sol_* = 30 mL; *m_adsorbent_* = 0.15 g for Co, MB and *I*_2_ and 0.30 g for Ni; *T* = 25 °C; *e_quilibrium_* = 24 h, *C*_0_ see Section 2.3).

**Table 3:** Nonlinear fitting parameters between *q_e_* and *C_e_* found using Langmuir adsorption model.

Element/Molecule	*q_m_*	*e (q_m_)*	*K_L_*	*e* (*K_L_*)	*R* ^2^
Nickel	22	3	0.0040	±0.0010	0.98
Cobalt	23	4	0.0015	±0.0001	0.92
Methylene Blue	250	40	0.063	±0.055	0.86
Iodine	2680	400	0.00001	±0.00001	0.89

(with *e*(*i*) the error on parameter *i*).

**Table 4:** Parameter results of *q_m_* and *K_L_* from the linear fitting between *C_e_/q_e_* and *C_e_* using modified Langmuir adsorption equation: $\frac{C_{e}}{q_{e}}=\;\frac{1}{q_{m}.K_{L}}+\left(\frac{1}{q_{m}}\right)\cdot C_{e}$.

Element/Molecule	*q_m_*	*e(q_m_)*	*K_L_*	*e(K_L_)*	*R* ^2^
Nickel	13	±3	5	±1	0.71
Cobalt	30	±4	3	±1	0.28
Methylene Blue	251	±9	5	±1	0.99
Iodine	2366	±3	12	±4	0.92

(with *e*(*i*) the error on parameter *i*).

**Table 5:** Langmuir separation factors.

Element/Molecule	*C_o_* (mg/L)	*R_L_*
Nickel	25–700	0.91–0.26
Cobalt	25–700	0.96–0.49
Methylene Blue	250–2000	0.059–0.008
Iodine	3585–74,438	0.96–0.57

**Table 6:** Parameter results from nonlinear fitting between *q_e_* and *C_e_* using Freundlich model (**a**); and parameter results from linear fitting between log(*q_e_*) and log(*C_e_*) using Freundlich model (**b**).

Element/Molecule	*K_F_*	*e*(*K_F_*)	*N*	*e*(*n*)	*R* ^2^
**(a)**					
Nickel	0.72	±0.14	0.51	±0.01	0.99
Cobalt	0.13	±0.04	1.00	±0.07	0.99
Methylene Blue	106	±10	0.13	±0.01	0.99
Iodine	53	±16	0.35	±0.02	0.99
**(b)**					
Nickel	0.44	±0.06	0.57	±0.01	0.99
Cobalt	0.19	±0.05	0.86	±0.05	0.99
Methylene Blue	89	±3	0.165	±0.008	0.99
Iodine	40	±3	0.377	±0.008	0.99

$q_{e}=K_{F}.C_{e}{}^{n}$ (with *e*(*i*) the error on parameter *i*); $\log q_{e}=n.\log C_{e}+\log K_{F}$ (with *e*(*i*) the error on parameter *i*).

**Table 7:** *q_e_* and *S_C_* parameters found at different energies.

**Nickel**			**Pixels in**	**“1”**			
	*q_e_* (mg/g)	*S_C_* (22 keV)	*S_C_* (27 keV)	*S_C_* (33 keV)	*S_C_* (40 keV)	*S_C_* (50 keV)	*S_C_* (60 keV)
	0	0	0	0	0	0	0
	1.46	3205	2	1	0	0	0
	2.92	6410	4	2	0	0	0
	5.09	12,365	371	4	0	0	0
	7.27	18,320	738	6	0	0	0
	11.51	19,186	1040	11	0	0	0
	15.73	20,052	1342	17	0	0	0
**Cobalt**			**Pixels in**	**“1”**			
	*q_e_* (mg/g)	*S_C_* (22 keV)	*S_C_* (27 keV)	*S_C_* (33 keV)	*S_C_* (40 keV)	*S_C_* (50 keV)	*S_C_* (60 keV)
	0	0	0	0	0	0	0
	2.01	418	25	2	0	0	0
	4.03	835	50	5	0	0	0
	7.62	7192	222	97	0	0	0
	11.21	13,550	395	190	0	0	0
	27.58	13,800	621	227	0	0	0
	43.95	14,050	848	265	0	0	0
**MB**			**Pixels in**	**“1”**			
	*q_e_* (mg/g)	*S_C_* (22 keV)	*S_C_* (27 keV)	*S_C_* (33 keV)	*S_C_* (40 keV)	*S_C_* (50 keV)	*S_C_* (60 keV)
	0	0	0	0	0	0	0
	49.97	3857	15	0	0	0	0
	99.94	7714	30	0	0	0	0
	145.31	11,607	51	0	0	0	0
	190.68	15,500	73	0	0	0	0
	224.99	21,315	637	0	0	0	0
	259.3	27,130	1201	15	0	0	0
**Iodine**			**Pixels in**	**“1”**			
	*q_e_* (mg/g)	*S_C_* (40 keV)	*S_C_* (50 keV)	*S_C_* (60 keV)	*S_C_* (70 keV)	*S_C_* (80 keV)	*S_C_* (90 keV)
	0	0	0	0	0	0	0
	451	31,545	34,810	13,525	0	0	0
	903	63,090	69,620	27,050	0	0	0
	1232	51,470	74,755	36,060	0	0	0
	1561	69,850	79,890	45,070	0	0	0
	2099	57,575	81,285	51,985	0	0	0
	2638	75,300	82,680	58,900	0	0	0

**Table 8:** Fitting parameters found for *q_e_* value versus *Sc* parameter (XRA) for each adsorbed molecule or ion.

**Nickel**					
Evaluated Parameter	*A (Pixels)*	*e(A)*	*B (Pixels·g/mg)*	*e(B)*	*R* ^2^
S_C_ (22 keV–125 mAs)	3027	±4	1326	±162	0.91
S_C_ (27 keV–125 mAs)	−98	±7	95	±8	0.98
S_C_ (33 keV–125 mAs)	−0.900	±0.001	1.03	±0.05	0.99
**Cobalt**					
Evaluated Parameter	*A (Pixels)*	*e(A)*	*B (Pixels·g/mg)*	*e(B)*	*R* ^2^
S_C_ (22 keV–125 mAs)	2533	±200	333	±10	0.81
S_C_ (27 keV–125 mAs)	36	±10	20	±2	0.97
S_C_ (33 keV–125 mAs)	25	±12	6	±1	0.89
**Methylene Blue**					
Evaluated Parameter	*A (Pixels)*	*e(A)*	*B (Pixels·g/mg)*	*e(B)*	*R* ^2^
S_C_ (22 keV–125 mAs)	−1481	±129	100	±8	0.98
S_C_ (27 keV–125 mAs)	−237	±23	4	±1	0.76
S_C_ (33 keV–125 mAs)	−2.56	±0.54	0.03	±0.02	0.56
**Iodine**					
Evaluated Parameter	*A (Pixels)*	*e(A)*	*B (Pixels·g/mg)*	*e(B)*	*R* ^2^
S_C_ (40 keV–40 mAs)	19,647	±160	24	±7	0.83
S_C_ (50 keV–40 mAs)	23,536	±122	29	±8	0.85
S_C_ (60 keV–40 mAs)	4498	±50	22	±2	0.98

$S_{C}=\;A+B.q_{e}$ (with *e*(*i*) the error on parameter *i*).

## Data Availability

Data sharing is not applicable to this article because of authorized issues.

## Supplementary Materials

The following are available online at https://www.mdpi.com/1996-1944/14/1/91/s1, Figure S1: Correlation graphs between *S_C_* and *q_e_* values (**a**) nickel, (**b**) cobalt, (**c**) MB and (**d**) Iodine for virgin GAC1 and ion or molecule loaded GAC1 samples.

## References

[1] Van Grieken R., Markowicz A. (2001). Handbook of X-ray Spectrometry.

[2] Depristo A.E., Augustin S.D., Ramaswamy R., Rabitz H. (1979). Quantum number and energy scaling for nonreactive collisions. J. Chem. Phys..

[3] Massimi M. (2005). Pauli’s Exclusion Principle: The Origin and Validation of a Scientific Principle.

[4] Gonzalez R.C., Woods R.E., Eddins S.L. (2004). Digital Image Processing Using MATLAB.

[5] Vanderheyden S.R.H., Yperman J., Carleer R., Schreurs S. (2018). Enhanced cesium removal from real matrices by nickel-hexacyanoferrate modified activated carbons. Chemosphere.

[6] Vanreppelen K., Vanderheyden S., Schreurs S., Yperman J., Băbeanu N., Carleer R. (2014). Activated carbon from pyrolysis of brewer’s spent grain: Production and adsorption properties. Waste Manag. Res..

[7] Veith J., Sposito G. (1977). On the use of the Langmuir equation in the interpretation of “adsorption” phenomena. Soil. Sci. Soc. Am. J..

[8] Harter R.D., Smith G. (1981). Langmuir equation and alternate methods of studying “adsorption” reactions in soils. Chem. Soil Environ..

[9] Tran H.N., You S.-J., Hosseini-Bandegharaei A., Chao H.-P. (2017). Mistakes and inconsistencies regarding adsorption of contaminants from aqueous solutions: A critical review. Water Res..

[10] Reed B.E., Matsumoto M. (1993). Modeling Cadmium Adsorption by Activated Carbon Using the Langmuir and Freundlich Isotherm Expressions. Sep. Sci. Technol..

[11] Johns H., Cunningham J. (1983). The Physics of Radiology.

[12] Kumar A. (2016). Studies on effective atomic numbers and electron densities of nucleobases in DNA. Radiat. Phys. Chem..

[13] Taylor M.L., Smith R.L., Dossing F., Franich R.D. (2012). Robust calculation of effective atomic numbers: The Auto-Zeff software. Med. Phys..

[14] Tonguc B.T., Arslan H., Al-Buriahi M. (2018). Studies on mass attenuation coefficients, effective atomic numbers and electron densities for some biomolecules. Radiat. Phys. Chem..

[15] Torres J.P., Sariol H.C., Yperman J., Sauvanell Á.B., Carleer R., Campa J.N. (2018). A novel X-ray radiography approach for the characterization of granular activated carbons used in the rum production. J. Anal. Sci. Technol..

[16] Torres J.P., Sariol H.C., Yperman J., Adriaensens P., Carleer R., Peacok T.M., Sauvanell Á.B., Thijssen E., Reggers G., Haeldermans T. (2019). X-ray absorption as an alternative method to determine the exhausting degree of activated carbon layers in water treatment system for medical services. Talanta.

