# Supplementary material for: X-ray Absorption (XRA): A New Technique for the Characterization of Granular Activated Carbons
Source: Materials (Basel). 2020 Dec 28;14(1):91. doi: 10.3390/ma14010091 (PMC7795858; doi:10.3390/ma14010091)
Supplement: Supplementary file 1 [file materials-14-00091-s001.pdf]

materials

MDPI

Article

X-ray Absorption (XRA): A New Technique for the Characterization of Granular Activated Carbons

Jeamichel Puente Torres [1,2], Harold Crespo Sariol [3], Thayset Mariño Peacok [3], Jan Yperman [4,*], Peter Adriaensens [4], Robert Carleer [4] and Ángel Brito Sauvanell [5]

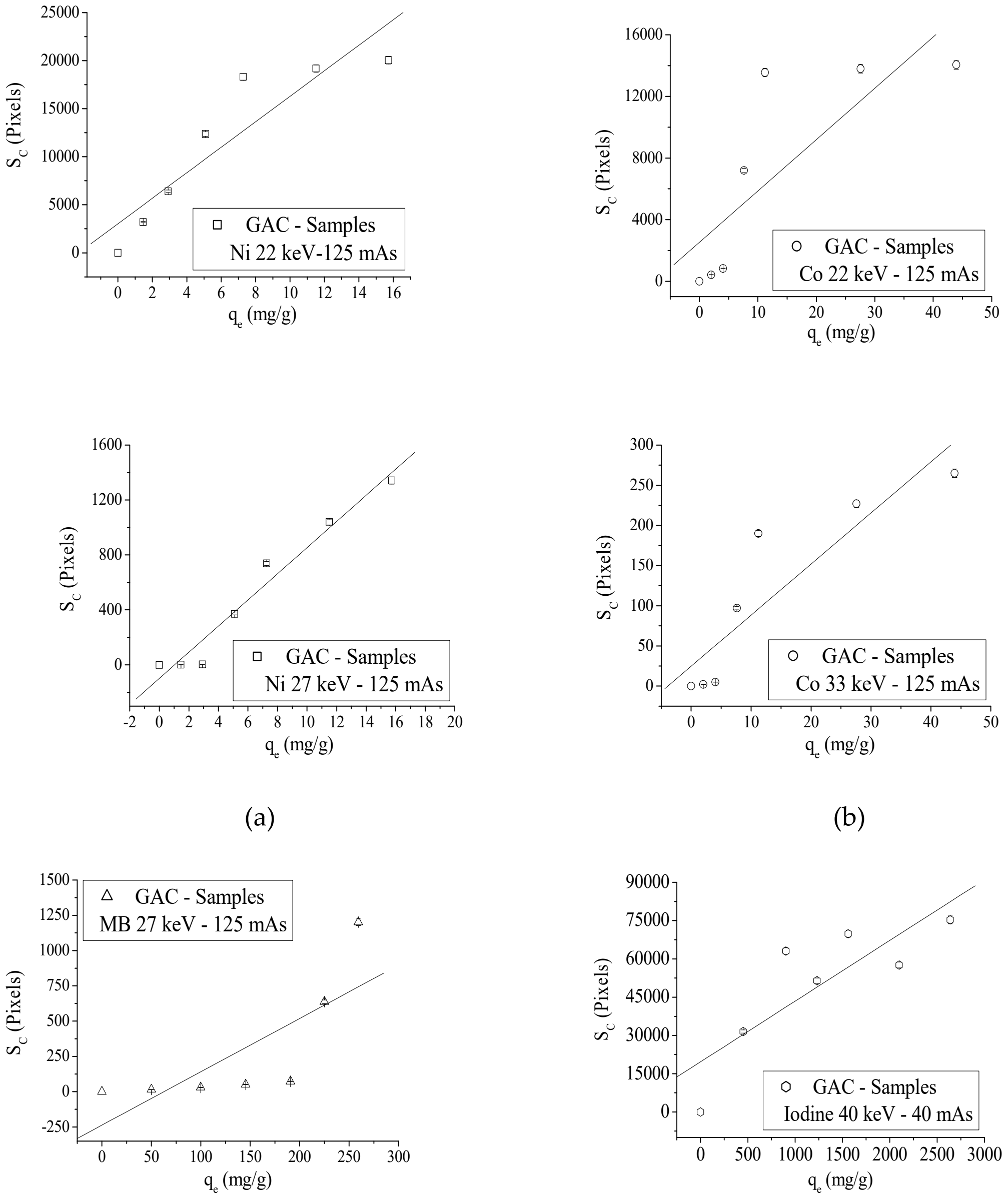

Materials **2021**, *14*, 91. https://doi.org/10.3390/ma14010091 www.mdpi.com/journal/materials

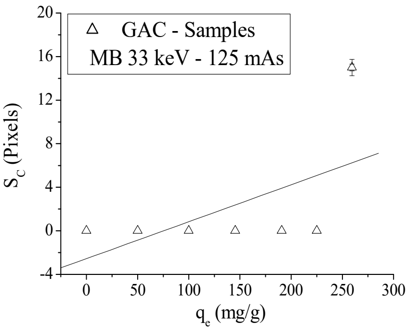

(c)

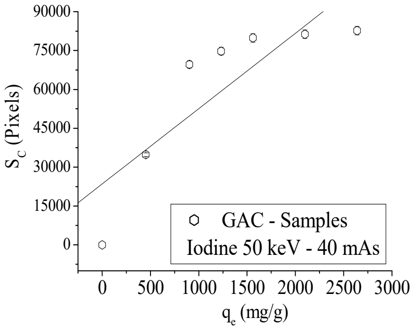

(d)

Figure S1. Correlation graphs between S_C and q_e values (**a**) nickel, (**b**) cobalt, (**c**) MB and (**d**) Iodine for virgin GAC1 and ion or molecule loaded GAC1 samples.
